# Prevention of Oxaliplatin-Induced Peripheral Neuropathy: A Systematic Review and Meta-Analysis

**DOI:** 10.3389/fonc.2022.731223

**Published:** 2022-02-03

**Authors:** Siyu Peng, Ariel Fangting Ying, Nicholas Jian Hao Chan, Raghav Sundar, Yu Yang Soon, Aishwarya Bandla

**Affiliations:** ^1^ Department of Medicine, National University Health System, Singapore, Singapore; ^2^ Health Services and System Research, Duke-National University of Singapore (NUS) Medical School, Singapore, Singapore; ^3^ Department of Haematology-Oncology, National University Cancer Institute Singapore, National University Health System, Singapore, Singapore; ^4^ The N.1 Institute for Health, National University of Singapore, Singapore, Singapore; ^5^ Yong Loo Lin School of Medicine, National University of Singapore, Singapore, Singapore; ^6^ Department of Radiation Oncology, National University Cancer Institute Singapore, National University Health System, Singapore, Singapore

**Keywords:** oxaliplatin, peripheral neuropathy, neurotoxicity, meta-analysis, pharmacological, non-pharmacological, network analysis

## Abstract

**Background:**

Oxaliplatin-induced peripheral neuropathy (OIPN) has significant clinical impact on the quality of life for cancer patients and is a dose limiting toxicity. Trials studying preventive measures have been inconclusive. A systematic review and meta-analysis were conducted to evaluate the existing pharmacological and non-pharmacological interventions to prevent chronic OIPN.

**Methods:**

Literature databases PubMed-MEDLINE, Embase and Scopus, were searched from 1 Jan 2005 to 08 Aug 2020 and major conferences’ abstracts were reviewed for randomized controlled trials that examined the efficacy of any preventive measure for OIPN. The primary outcome measure was the incidence of chronic OIPN with a preventive intervention as compared to placebo or no intervention. The pooled risk ratio and its 95% confidence interval were calculated using a random effects model. A network meta-analysis was conducted to derive indirect evidence of any preventive effect of an intervention against placebo when original trials compared one intervention against another.

**Results:**

Forty-four trials were analyzed describing 29 chemoprotective interventions, including combinations, and 1 non-pharmacological intervention. Ratings were assessed *via* a combination of outcomes with quality assessment using the Grading of Recommendations, Assessment, Development and Evaluations (GRADE) framework. Of the 30 interventions examined, there were six interventions supporting potential efficacy, 11 interventions with insufficient evidence and 13 interventions not recommended.

**Conclusion:**

Currently, there is insufficient certainty to support any intervention as effective in preventing OIPN. Of note is that most of these studies have focused on pharmacological interventions; non-pharmacological interventions are underexplored. Further research on ways to limit OIPN is needed.

**Systematic Review Registration:**

https://www.crd.york.ac.uk/prospero/display_record.php?RecordID=225095, Prospero Registration Number: CRD42021225095.

## Introduction

Oxaliplatin, a platinum-based anti-tumor agent, is a mainstay of treatment for many gastrointestinal cancers, particularly colorectal cancer, the second highest cause of cancer mortality worldwide (1). However, as many as 40-50% of patients receiving this drug will develop oxaliplatin-induced peripheral neuropathy (OIPN) (2, 3). While the acute form of OIPN is largely reversible within a week, about 20-50% of patients experience severe chronic OIPN which is dose-dependent, potentially persistent and debilitating (4–6). This high incidence of chronic neurotoxicity has significant impact on treatment continuity and quality of life in cancer patients and survivors, making OIPN an area of significant clinical importance (7, 8).

Many mechanisms for OIPN have been postulated, ranging from direct neurotoxicity from deoxyribonucleic acid (DNA) damage to ion channel dysfunction to oxidative stress (9, 10). As such, pre-clinical studies on OIPN prevention have examined the efficacy of a wide range of potential interventions, from neuro-active pharmaceuticals like gabapentin (11) and antioxidants like glutathione (12), to herbal medications like Goshajinkigan (13). However, attempts to translate encouraging results from animal studies to humans have met with limited success.

Although many clinical trials have attempted to identify interventions that can prevent OIPN in humans, the small sample size in most of these trials and the lack of standardized measurement metrics have rendered results difficult to interpret (14). Indeed, the latest oncology guidelines on chemotherapy-induced peripheral neuropathy (CIPN) acknowledge that despite the multitude of trials available, there is no convincing evidence that there is any intervention that can effectively prevent CIPN (7, 8).

In recent years, there has been an emerging interest in examining the usage of non-pharmacological interventions to prevent CIPN, such as cryotherapy, acupuncture, or exercise (15–18). One area that has drawn particular interest is the usage of cryotherapy, which has yielded promising results in taxane-induced peripheral neuropathy (16, 19, 20). Subsequent trials have recently begun looking into the efficacy of cryotherapy on OIPN as well (21).

Previous systematic reviews and meta-analyses have attempted to synthesize all the information available on CIPN, but they either focused on one specific intervention (22, 23) and/or were not specific to oxaliplatin (7, 8, 24). Given that there are distinct differences in the mechanism and clinical presentation of neuropathy induced by different chemotherapy agents, and between acute and chronic OIPN, evidence that is specific to chronic OIPN might better inform clinical practice (7, 25, 26).

This current systematic review/meta-analysis was conducted to determine the efficacy of both pharmacological and non-pharmacological preventive measures for chronic OIPN in patients with cancers treated with oxaliplatin-based chemotherapy.

## Methods

### Search Strategy

The systemic review and meta-analysis followed the Preferred Reporting Items for Systematic Reviews and Meta-analyses (PRISMA) guidelines (27). Two investigators (SP and AFY) independently searched, without language restriction, PubMed-MEDLINE, Embase and Scopus for randomized controlled trials (RCTs) published since 1 Jan 2005 to 8 Aug 2020. A detailed list of the search terms can be found in the **Supplementary Material**.

### Selection Criteria

Human RCTs that assessed the efficacy of any form of protective measure to try to prevent and/or reduce the incidence of chronic OIPN as compared with placebo, no intervention or other interventions were included. These trials must have enrolled adult cancer patients with the planned chemotherapy regimen containing oxaliplatin as the only neurotoxic chemotherapeutic agent and reported the incidence and/or severity of chronic OIPN measured using clinical scales or electrophysiological studies. Trials were excluded if they were single-armed, retrospective, non-randomized, enrolled patients with pre-existing peripheral neuropathy, enrolled patients not naïve to chemotherapy, did not report outcomes specific to OIPN, or only reported acute OIPN outcomes. The primary outcome was the incidence of grade 2 and above peripheral neuropathy as assessed by the Common Terminology Criteria for Adverse Events (CTCAE). Information on other outcomes such as neuropathy grades assessed by other neuropathy scales or electrophysiology measurements were also extracted. For trials that did not use CTCAE in the outcome assessment, the primary outcome measures reported by trials were used for analysis. For the trials that did not report the full details of outcomes for analysis, the authors were contacted for more information. If such data were not available, then such trials were excluded from analysis.

### Data Extraction

The same investigators (SP and AFY) independently extracted data from the selected trials. Discrepancies were resolved by consensus amongst both investigators and advice from a third independent investigator (RS). Information extracted included: intervention dose and route, nature of the control (placebo, no additional intervention, or another intervention), trial primary and secondary outcomes and their assessment methods (clinical neuropathy scales or neurological electrophysiological studies) and time of assessment, follow up duration, and cumulative dose of oxaliplatin. Information was also collected for trial quality assessment, as outlined below.

### Quality Assessment

Quality of the trials was assessed using the Risk of Bias Tool in Review Manager version 5.3 (RevMan 5.3) software by Nordic Cochrane Centre, and scored according to the domains of selection bias, performance bias, detection bias, attrition bias and reporting bias. The risk of bias results were scored on three levels, namely “high bias”, “low bias” and “unclear bias”, and then included into a rating approach proposed by the Grading of Recommendations, Assessment, Development and Evaluation (GRADE) Working Group to evaluate the certainty of the result across four domains, namely risk of bias, inconsistency, indirectness and imprecision (28). The final GRADE scores represent four levels of certainty, namely “very low” (1), “low” (2), “moderate” (3) and “high” (4) (**Supplementary Material**)

### Data Analysis

For trials using dichotomous outcome measures, the treatment effect was calculated across trials in the form of Risk Ratio (RR) based on the intention-to-treat sample size. If there were two or more RCTs that reported the same outcome measure by comparing the same combination of interventions, the respective RR were summarized in Forest plots using the random effects model of meta-analysis, in RevMan 5.3. All reported *p*-values were two-sided and *p*<0.05 indicated statistical significance. Heterogeneity was assessed *via* Cochran’s Q test and Higgins’ I^2^ test. If significant methodological or statistical heterogeneity was detected, sensitivity analysis was performed based on factors that contributed to heterogeneity. For continuous outcomes, such as pain or neuropathy scores, the difference in means was calculated and the results were expressed with 95% confidence intervals (CI). For outcomes that did not fit the above criteria, significant outcomes were summarized as they were presented in the results section of the original trial.

A network meta-analysis was also conducted using R programming (*netmeta* package, frequentist method and random effects model), to obtain indirect evidence of treatment effect from the trials that compared two types of interventions. The main purpose of performing a network meta-analysis is to be able to derive indirect comparisons between a type of intervention and control (either placebo or no additional intervention), as such information was not available from the original trials (29).

## Results

### Trial Selection

**Figure 1:**
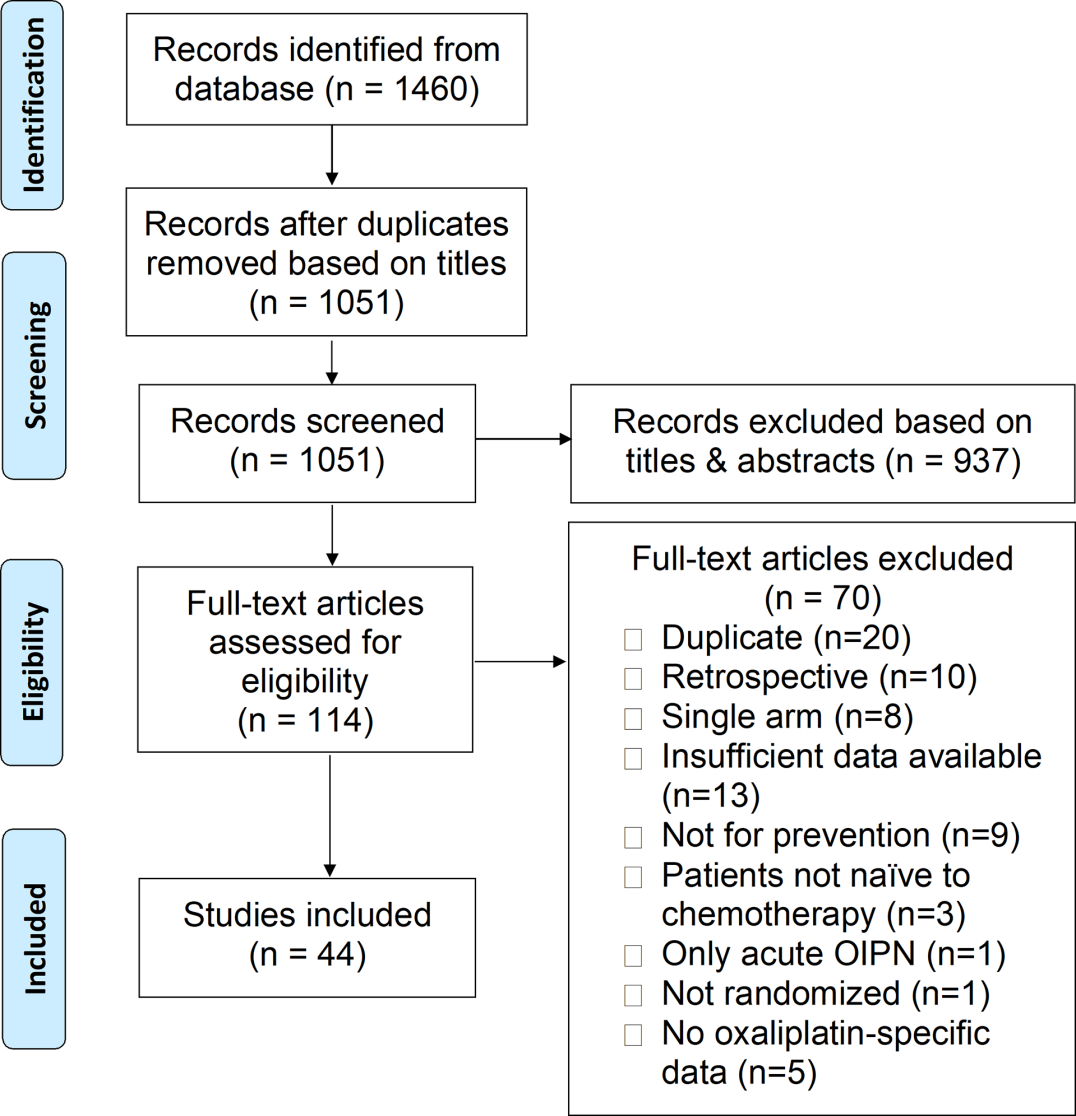
PRISMA flow diagram.

The search strategy (**Figure 1**) derived a total of 44 RCTs that examined 30 interventions for OIPN prevention, of which 43 trials examined 29 pharmacological interventions and 1 trial examined 1 non-pharmacological interventions. Of those that compared pharmacological interventions against placebo or no additional intervention, seven trials examined calcium and magnesium (Ca/Mg) infusions, ten examined neuro-active pharmaceuticals, six examined other pharmaceuticals, eleven examined antioxidants, and seven examined herbal medicines. One of the trials, Dong et al. (30), assessed two different types of interventions; thus, it was classified under two sections. In addition, three trials compared two types of interventions (or combination interventions) and their results were analyzed separately using network meta-analysis.

Among the excluded studies, seven RCTs that enrolled patients receiving oxaliplatin, and whose primary outcome was the prevention of chronic CIPN, were also identified *via* full text screening. Of these, two trials (31, 32) enrolled patients with prior platinum exposure and the remaining five (21, 33–36) did not provide sufficient data for analysis. Hence, all seven of these trials were excluded from the final analysis.

### Rating Criteria for Interventions

**Figure 2:**
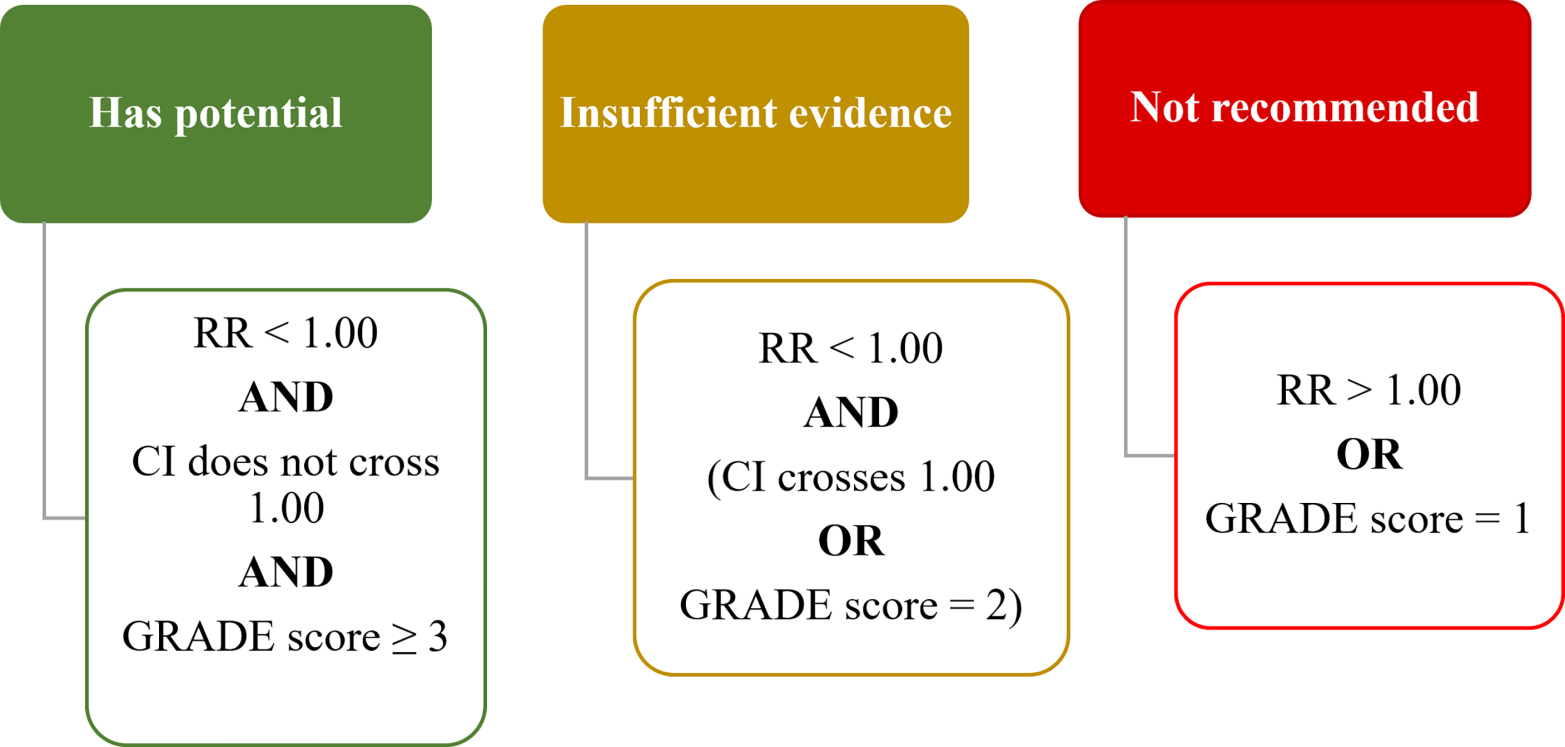
Rating criteria on interventions to prevent OIPN. RR, risk ratio; CI, confidence interval. GRADE score ranges from High (4+), Moderate (3+), Low (2+) to Very Low (1+). (RR, Risk Ratio; CI, Confidence Interval).

Based on the RR calculated from primary outcome measures and the GRADE evaluation of certainty of evidence, the interventions were assigned to one of three levels of recommendation, namely “Has Potential”, “Insufficient Evidence” and “Not Recommended” (**Figure 2**). An intervention was rated to have “Potential” if the RR < 1.00, the CI did not cross 1.00, and the GRADE evaluation score was ≥ 3 (“moderate” or “high”). An intervention was rated to have “Insufficient Evidence” if the RR < 1.00, but either the CI crossed 1.00 or the GRADE evaluation score was = 2 (“low”). An intervention was rated to be “Not Recommended” if the RR > 1.00 or the GRADE evaluation score was = 1 (“very low”). When there was more than one outcome measure reported, the primary outcome measures using CTCAE was prioritized in the overall evaluation, while evaluations based on other outcome measures were recorded in the **Table S1** and the scales explained in **Table S2**. In summary, six interventions were rated as having “Potential”, nine interventions as “Insufficient Evidence” and fifteen interventions as “Not Recommended”.

In addition, among the fourteen interventions with statistically significant lower RR of OIPN incidence, including L-carnosine (**Figure S1**), six were classified as “insufficient evidence” while two were classified as “not recommended” as their overall ratings were limited by “low” and “very low” GRADE certainties respectively (**Table 1**)

**Table 1:** Categories of recommendation.

Intervention	Outcome	No. of studies	No. of patients	RR (95% CI)	Certainty	Risk of bias	Inconsistency	Indirectness	Imprecision
**Has Potential**									
AC591 (37)	Levi >= Grade 2	1	72	RR 0.09 (0.01, 0.67) *	High (4+)	Not serious	Not serious	Not serious	Not serious
Ca/Mg (3, 30, 38–41)	CTCAE^ >= Grade 2	6	613	RR 0.74 (0.55, 0.99) *	Moderate (3+)	Serious	Not serious	Not serious	Not serious
Glutathione (GSH) (30, 42, 43)	CTCAE >= Grade 2	3	159	RR 0.50 (0.36, 0.72) *	High (4+)	Not serious	Not serious	Not serious	Not serious
Guilongtongluofang (44)	CTCAE Grade 3-4	1	120	RR 0.37 (0.17, 0.81) *	High (4+)	Not serious	Not serious	Not serious	Not serious
N3 PUFA (45)	Reduced TNS^†^ incidence	1	71	RR 0.60 (0.43, 0.83) *	High (4+)	Not serious	Not serious	Not serious	Not serious
N-acetylcysteine (NAC) (46, 47)	CTCAE >= Grade 2	2	46	RR 0.26 (0.12, 0.56) *	Moderate (3+)	Serious	Not serious	Not serious	Not serious
**Insufficient Evidence**									
Carbamazepine (48)	Levi Grade 3-4	1	36	RR 0.60 (0.20,1.76)	Low (2+)	Serious	Not serious	Not serious	Serious
Glutamine (49)	CTCAE Grade 3-4	1	86	RR 0.37 (0.15, 0.95) *	Low (2+)	Very serious	Not serious	Not serious	Not serious
L-carnosine (50, 51)	CTCAE >= Grade 2	2	121	RR 0.05 (0.01, 0.22) *	Low (2+)	Very serious	Not serious	Not serious	Not serious
Metformin (52)	CTCAE >= Grade 2	1	40	RR 0.63 (0.44, 0.92) *	Low (2+)	Very serious	Not serious	Not serious	Not serious
Neurotropin (53)	CTCAE >= Grade 2	1	79	RR 0.35 (0.18, 0.67) *	Low (2+)	Very serious	Not serious	Not serious	Not serious
Ninjin’yoeito (54)	CTCAE >= Grade 2	1	52	RR 0.18 (0.04, 0.74) *	Low (2+)	Very serious	Not serious	Not serious	Not serious
Oxcarbazepine (55)	Modified TNS^†^incidence	1	40	RR 0.42 (0.18, 0.96) *	Low (2+)	Serious	Not serious	Not serious	Not serious
Pregabalin (56)	Brief pain inventory	1	143	Continuous, non-significant benefit	Low (2+)	Serious	Not serious	Not serious	Serious
Venlafaxine (57, 58)	CTCAE >= Grade 2	2	101	RR 0.75 (0.47, 1.19)	Moderate (3+)	Not serious	Not serious	Serious	Serious
**Not Recommended**									
Air wave pressure (59)	CTCAE >= Grade 2	1	45	RR 0.19 (0.02, 1.51)	Very low (1+)	Very serious	Not serious	Not serious	Serious
Alpha-lipoic acid (ALA) (60)	NCI Sanofi^‡^ >= Grade 2	1	49	RR 0.69 (0.39, 1.23)	Very low (1+)	Very serious	Not serious	Not serious	Serious
Amifostine (49, 61)	CTCAE Grade 3-4	2 (NMA)	173	RR 0.02 (0.00, 0.48) *	Very low (1+)	Serious	Not serious	Serious	Serious
ART-123 (62)	CTCAE >= Grade 2	1	42	RR 1.40 (0.84, 2.35)	Low (2+)	Serious	Not serious	Not serious	Serious
Calmangafodipir (63)	OSSS^‡^ Grade 2-3	1	173	RR 0.61 (0.32, 1.16)	Very low (1+)	Very serious	Not serious	Serious	Serious
Cystine & theanine (64)	Mean CTCAE score	1	28	Continuous, reported as significant benefit *	Very low (1+)	Very serious	Not serious	Serious	Not serious
Glutamine + Ca/Mg (3, 30, 38–41, 65)	CTCAE >= Grade 2	7 (NMA)	813	RR 0.76 (0.43, 1.35)	Very low (1+)	Very serious	Not serious	Serious	Serious
Ganglioside-monosialic acid (GM1) (66)	CTCAE >= Grade 2	1	192	RR 1.07 (0.71, 1.61)	Moderate (3+)	Not serious	Not serious	Not serious	Not serious
Goshajinkigan (GJG) (67, 68)	CTCAE >= Grade 2	2	271	RR 1.12 (0.53, 2.37)	Very low (1+)	Serious	Serious	Not serious	Serious
Minocycline (69)	MDASI AUC^#^	1	66	Continuous, non-significant harm	Moderate (3+)	Not serious	Not serious	Not serious	Serious
MR309 (70)	CTCAE >= Grade 2	1	87	RR 1.18 (0.91, 1.52)	Low (2+)	Serious	Not serious	Serious	Serious
Riluzole (71)	Reduced TNS^†^ incidence	1	48	Continuous, significant harm	High (4+)	Not serious	Not serious	Not serious	Not serious
Tanshinone IIA (72)	Levi’s Grade 3-4	1	36	RR 5.00 (0.26, 97.37)	Very low (1+)	Very serious	Not serious	Not serious	Serious
Vitamin E (73)	Neuropathy incidence	1	65	RR 1.03 (0.95, 1.12)	Very low (1+)	Very serious	Not serious	Not serious	Serious
Vitamin E + Ca/Mg (3, 30, 38–41, 74)	CTCAE >= Grade 2	7 (NMA)	647	RR 1.98 (0.22, 17.95)	Very low (1+)	Not serious	Not serious	Serious	Serious

Total 30 interventions were classified into three levels of recommendation, namely “have potential”, “insufficient evidence and “not recommended”.

CTCAE^, Common Terminology Criteria for Adverse Events; TNS^†^, Total Neuropathy Score; NCI Sanofi/OSSS^‡^, National Cancer Institute Sanofi scale, also known as Oxaliplatin Sanofi Specific Scale; MDASI AUC^#^, MD Anderson Symptom Inventory (MDASI) score for numbness/tingling and fatigue, calculated as Area Under Curve; Risk ratios marked with * indicates that they are statistically significant where 95% confidence interval is less than 1.

Refer to the **Supplementary Material** for summaries of trial characteristics and risk of bias of all trials (**Table S3**), dichotomous or continuous outcome results analyzed from single trials (**Tables S4**, **S5**) and table presentation of network meta-analysis results (**Table S6**).

### Type of Intervention

For the ease of presentation, the pharmacological interventions were classified into five groups based on mechanisms of action, namely Ca/Mg, neuro-active pharmaceuticals, other pharmaceuticals, antioxidants, and herbal medicine.

#### 1. Calcium and Magnesium (Ca/Mg)

A total of seven trials examined the effect of intravenous Ca/Mg infusions versus placebo on the prevention of OIPN (3, 30, 38–41, 75). Of these, only Gobran et al. (40) reported a statistically significant reduction in OIPN incidence using Chi-square test.

All seven trials were double-blinded and used placebo saline infusions for the control group (**Table S3**). However, three of the trials, namely the ones by Ishibashi et al. (39), Chay et al. (75), and Grothey et al. (41), were terminated early due to an interim report from the Combined Oxaliplatin Neurotoxicity Prevention Trial (CONcePT) (76) in 2007, which suggested that Ca/Mg infusion could worsen response to chemotherapy. In addition, two of the other trials, by Gobran et al. (40) and Dong et al. (30), had high attrition rates of ≥30%. Furthermore, the trial by Dong et al. (30) only reported the incidence of neuropathy at each assessment point, instead of the cumulative incidence.

**Figure 3:**
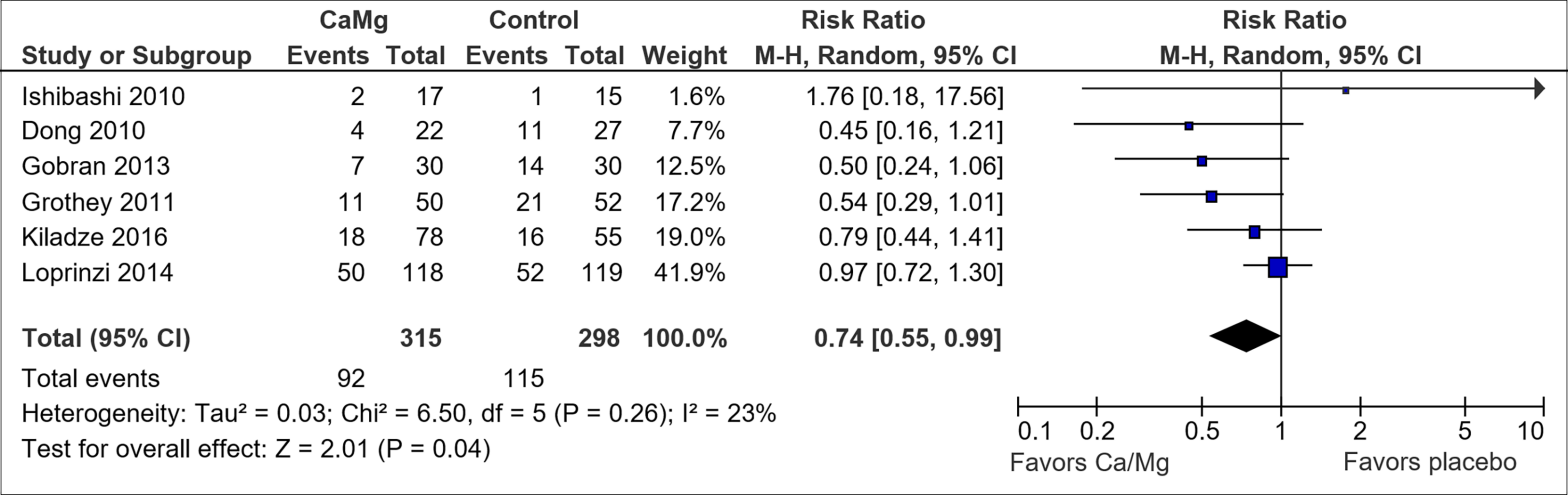
Forest plot for comparison of the incidence of OIPN of CTCAE grade ≥ 2: Ca/Mg versus placebo.

Six of the trials used CTCAE to assess outcome and their results were pooled in a Forest plot (**Figure 3**) (3, 30, 38–41). Ca/Mg infusion was associated with a statistically significant reduction in the risk of CTCAE grade 2 and above neuropathy (RR, 0.74; 95% CI, 0.55-0.99). However, after performing sensitivity analysis to exclude the two trials with early termination (39, 41), pooled results were no longer statistically significant (RR, 0.76; 95% CI, 0.54-1.08) (**Figure S2**). Pooled analysis from three trials (3, 41, 75) on the secondary outcome also showed non-statistically significant reduction in the incidence of OIPN of Oxaliplatin Specific Scale (OSS) grade ≥ 2 (RR, 0.76; 95% CI, 0.56-1.03) (**Figure S3**). Symmetrical funnel plot (**Figure S4**) showed low publication bias.

#### 2. Neuro-Active Pharmaceuticals

A total of ten trials examined the effect of neuro-active pharmaceuticals versus placebo on the prevention of OIPN. Of these, four of them studied anti-epileptics (carbamazepine, oxcarbazepine, pregabalin and riluzole) (48, 55, 56, 71), four studied pain medications (ganglioside-monosialic acid, Novel Sigma-1 Receptor Antagonist MR309, and neurotropin) (53, 66, 70, 77), and the remaining two studied the antidepressant venlafaxine (57, 58) (**Table S3**).

None of the neuro-active pharmaceuticals showed potential benefit (**Table 1**)

#### 3. Other Pharmaceuticals

A total of six trials examined the effect of other pharmaceuticals versus placebo on the prevention of OIPN. There were two trials on N-acetylcysteine (46, 47), one on calmangafodipir (63), one on metformin (52), one on minocycline (69), and one on thrombomodulin (62) (**Table S3**).

**Figure 4:**
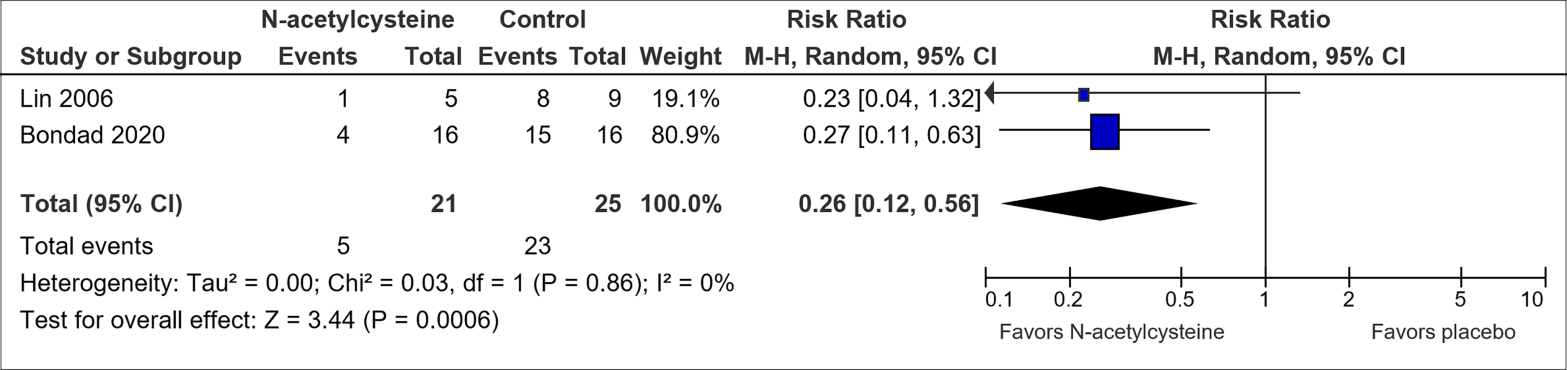
Forest plot for comparison of the incidence of OIPN of CTCAE grade ≥ 2: N-acetylcysteine versus placebo.

Only N-acetylcysteine showed potential for OIPN prevention (**Table 1**). Pooled results suggested an association between N-acetylcysteine and a reduced risk of CTCAE grade 2 and above neuropathy (RR, 0.26; 95% CI, 0.12-0.56) (**Figure 4**). However, the pooled sample size is small (n=46). In addition, the trial by Lin et al. (46) did not utilize blinding or placebo and hence further studies are warranted.

#### 4. Antioxidants

A total of eleven trials examined the effect of antioxidants versus placebo on the prevention of OIPN. These included one trial on alpha lipoic acid (60), two on glutamine (49, 78), three on reduced glutathione (30, 42, 43), two on L-carnosine (50, 51), one on vitamin E (73), one on n-3 polyunsaturated fatty acids (n-3 PUFA) (45), and one on cystine plus theanine (64) (**Table S3**).

**Figure 5:**
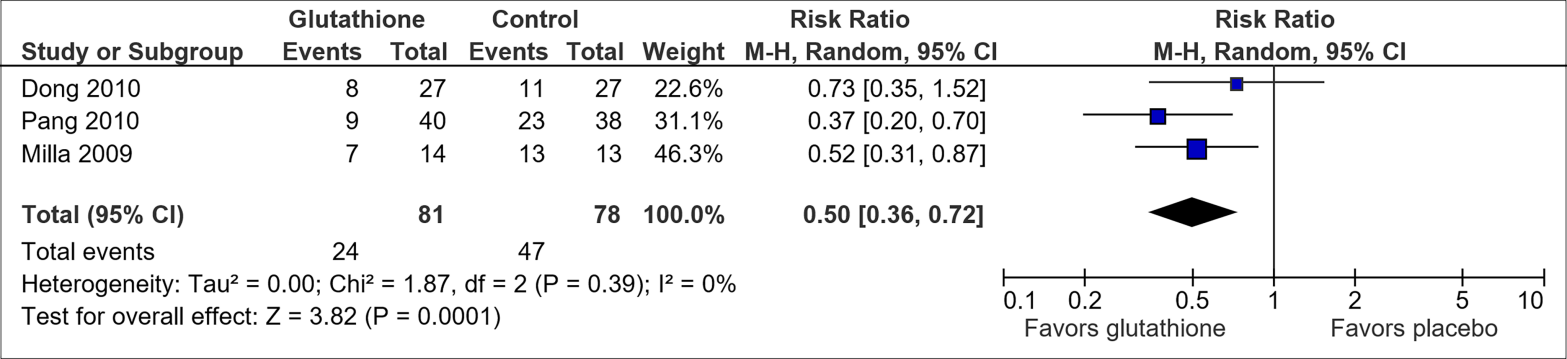
Forest plot for comparison of the incidence of OIPN of CTCAE grade ≥ 2: Reduced glutathione versus placebo.

Reduced glutathione was assessed to have potential benefit in preventing OIPN (**Table 1**). Pooled results from the three trials on reduced glutathione (30, 42, 43) suggested an association between reduced glutathione and a lowered risk of CTCAE grade 2 and above neuropathy (RR, 0.50; 95% CI, 0.36-0.72) (**Figure 5**). The overall certainty of the trials was high. However, the trial by Dong et al. (30) had a high attrition rate of 47.3% by cycle 9 (20% by cycle 6). The trial by Milla et al. *(* 42) and Pang et al. (43) used placebo but did not mention if allocation concealment or blinding was utilized.

N-3 PUFA also showed potential benefit in preventing OIPN. The only trial on n-3 PUFA by Esfahani et al. (45) showed a reduction in the risk of neuropathy as measured by the reduced Total Neuropathy Score (RR, 0.60; 95% CI, 0.43-0.83) (**Table 1**). However, this benefit was not consistently observed in secondary outcome measured in sensory nerve conduction studies and the trial was limited by the suboptimal placebo use (placebo did not smell like the intervention), small sample size and short follow-up period, thus requiring further studies to affirm the result.

#### 5. Herbal Medicines

A total of seven trials examined the effect of herbal medicines versus placebo on the prevention of OIPN. There were three trials on goshajinkigan (67, 68, 79), one on Guilongtongluofang (44), one on ninjin’yoeito (54), one on Tanshinone IIA (72), and one on AC591 (37) (**Table S3**). Only AC591 and guilongtongluofang were rated to “have potential” in reducing OIPN risk (**Table 1**).

AC591 was associated with a reduction in grade 2 and above neuropathy assessed *via* Levi’s scale (RR, 0.09; 95% CI, 0.01-0.67) (37). However, participants were only followed up for two months, i.e., four cycles of chemotherapy.

Guilongtongluofang was associated with a reduction in CTCAE grade 3-4 neuropathy (RR, 0.37; 95% CI, 0.17-0.81) (44). This trial had a low risk of bias in all categories. Patients received six cycles of chemotherapy and were followed up for two months thereafter.

#### 6. Non-Pharmacological Interventions

Only one trial examined the effect of non-pharmacological interventions on the prevention of OIPN (**Table S3**). In their trial on air wave pressure, Qian et al. (59) did not find any association with OIPN incidence (RR, 0.19; 95% CI, 0.02-1.51) (**Table 1**). Due to the nature of the intervention, placebo usage and thus blinding was not possible.

#### 7. Network Meta-Analysis

In order to assess treatment effect in the trials that compared two different interventions or intervention combinations, network meta-analysis was performed to link these trials with other related trials. There were three such trials (61, 65, 74) (**Table S3**). Network graphs (**Figure S5**) illustrate the type of comparison between different interventions. The presence of lines between a pair of nodes (interventions) indicates direct comparison from original trials, while the absence of a line indicates indirect comparison derived from network meta-analysis (29). The thickness of the lines is proportional to the number of trials that studied the respective direct comparison.

**Figure 6:**
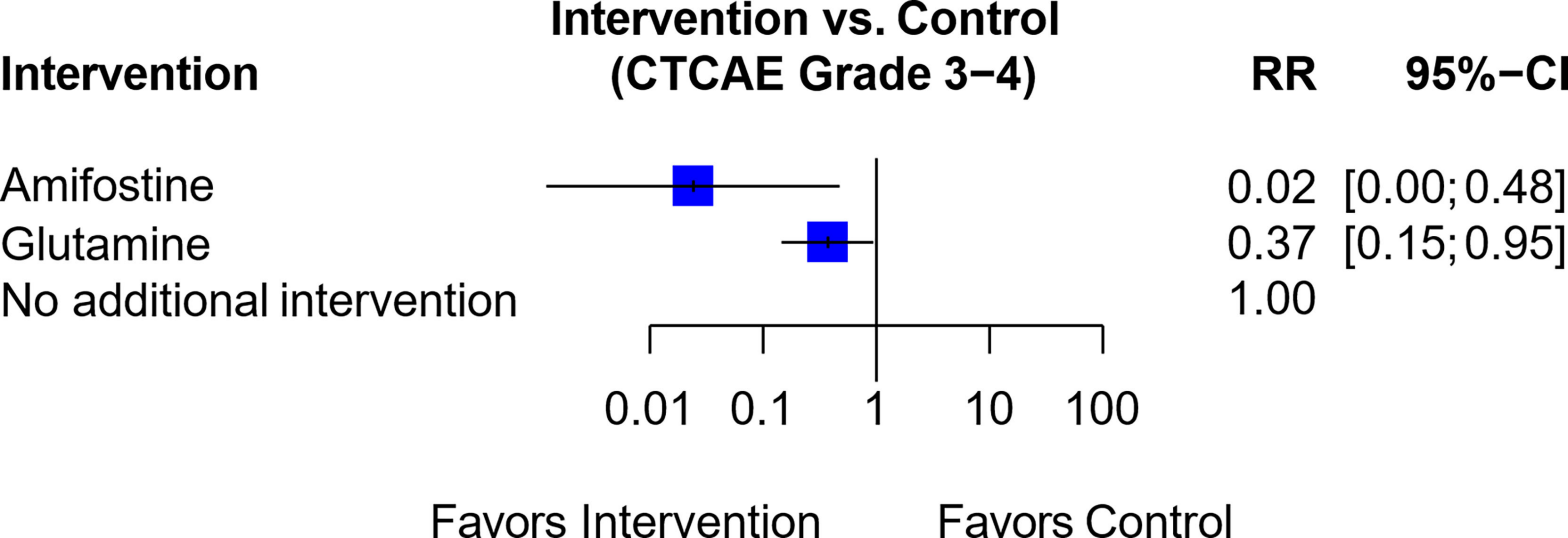
Forest plot for network analysis comparing Amifostine, Glutamine and control (no additional intervention), on the risk of CTCAE Grade 3-4 OIPN. Risk ratio comparing amifostine to no additional intervention was derived indirectly from network meta-analysis, while the risk ratio comparing glutamine against no additional intervention was derived directly from original trial data.

The trial by Lu et al. assessed the efficacy of amifostine versus glutamine (61). This was linked to a trial by Wang et al., which compared glutamine against a control group that received no additional intervention (49) (**Figure S5A**). The resultant network analysis (**Figure 6**) suggested that both amifostine (RR, 0.02; 95% CI, 0.00-0.48) and glutamine (RR, 0.37; 95% CI, 0.15-0.95) were associated with a reduced risk of Grade 3-4 OIPN as compared to no additional intervention. However, the overall GRADE certainty for the two studies involved was “very low (1+)”, and hence amifostine was rated “Not Recommended” (**Table 1**).

**Figure 7:**
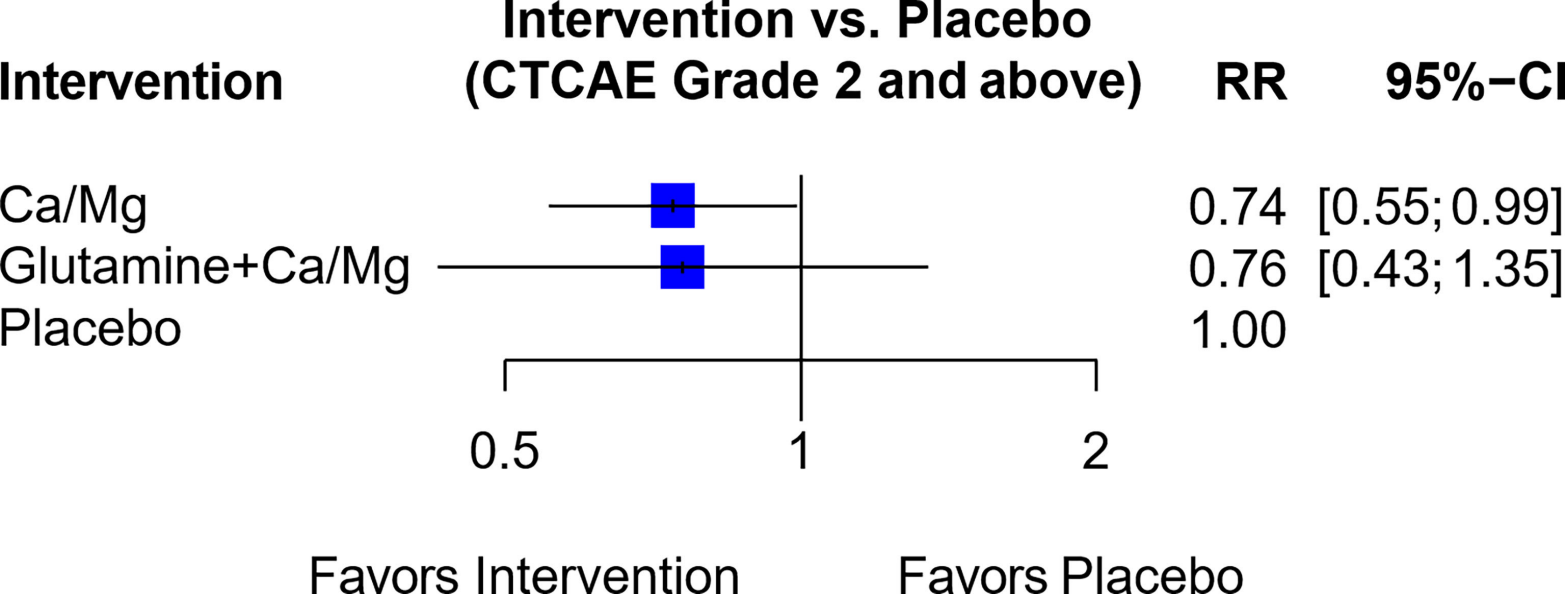
Forest plot for network analysis comparing Glutamine + Ca/Mg, Ca/Mg and placebo on the risk of OIPN of CTCAE grade ≥ 2. Risk ratio comparing combination therapy “glutamine + Ca/Mg” against placebo was derived indirectly from network meta-analysis, while the risk ratio comparing Ca/Mg against placebo was derived directly from 6 trials that conducted the exact comparison.

In a network of seven trials (**Figure S5B**), six trials assessed Ca/Mg versus placebo (3, 30, 38–41) while one trial assessed a combination of glutamine and Ca/Mg versus Ca/Mg alone (65). The resultant RR comparing the combination of glutamine and Ca/Mg versus placebo was not statistically significant (RR, 0.76; 95% CI, 0.43-1.35) (**Figure 7**), and the trial by Samson et al. (65) also used an open label design instead of blinding. Hence, the combination of glutamine plus Ca/Mg was rated “Not Recommended” (**Table 1**).

**Figure 8:**
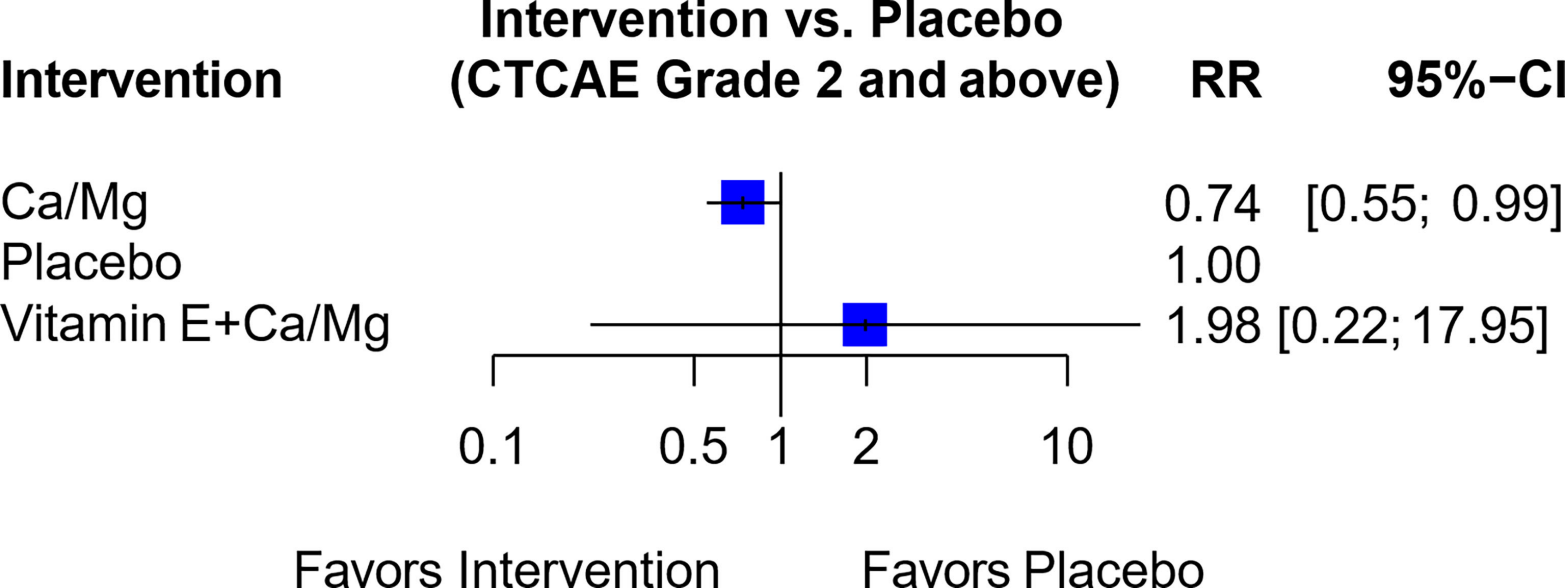
Forest plot for network analysis comparing Vitamin E + Ca/Mg, Ca/Mg and placebo on the risk of OIPN of CTCAE grade ≥ 2. Risk ratio comparing combination therapy “Vitamin E+ Ca/Mg” against placebo was derived indirectly from network meta-analysis, while the risk ratio comparing Ca/Mg against placebo was derived directly from 6 trials that conducted the exact comparison.

In another network of seven trials (**Figure S5C**), six trials compared Ca/Mg versus placebo (3, 30, 38–41) while one trial compared the combination of vitamin E and Ca/Mg versus Ca/Mg alone (74). This combination of vitamin E and Ca/Mg was also rated “Not Recommended” since the indirect comparison showed RR of 1.98 (95% CI, 0.22-17.95) between the combination of Vitamin E and Ca/Mg against placebo (**Figure 8** and **Table 1**).

## Discussion

This meta-analysis provides a focused update on existing evidence of OIPN prevention methods, including pharmacological and non-pharmacological interventions. Thirty interventions were analyzed comparing the risk ratio measured using clinical scales or electrophysiological testing. For the ease of presentation, the pharmacological interventions were classified into five groups, namely Ca/Mg, neuro-active pharmaceuticals, other pharmaceuticals, antioxidants, and herbal medicine.

Based on outcome measures and the GRADE evaluation of certainty of evidence, the interventions were assigned to one of three levels of recommendation, namely “Has Potential”, “Insufficient Evidence” and “Not Recommended” (**Figure 2**). With these criteria, six interventions that may have potential were identified, namely Ca/Mg, glutathione, n-3 PUFA, N-acetylcysteine, AC591 and Guilongtongluofang. Apart from the interventions highlighted in the latest recommendations from American Society of Clinical Oncology (ASCO) and European Society for Medical Oncology (ESMO) (7, 8), this study included thirteen additional interventions. While the assessment of interventions in the “Insufficient Evidence” and “Not Recommended” categories were consistent with the guidelines, further six interventions identified as “Has Potential” warrant further investigation (**Table 1**).

While the pooled results from six RCTs showed that Ca/Mg was associated with a statistically significant reduction in CTCAE grade 2 and above (**Figure 3**), this benefit was no longer observed following a sensitivity analysis that excluded two studies with early termination (39, 41) (**Figure S2**). In addition, the pooled results for studies that assessed outcome *via* OSS grade 2 and above neuropathy was not statistically significant (**Figure S3**). Hence, it is not possible to conclude from these results that Ca/Mg is effective in preventing OIPN.

Three other pharmaceuticals, namely N-acetylcysteine, glutathione and n-3 PUFA, appeared to have the potential to prevent OIPN. While N-acetylcysteine was associated with a statistically significant reduction in CTCAE grade 2 and above neuropathy based on pooled results from two trials (46, 47) (**Figure 4**), the overall small sample size and lack of blinding of one trial limited the overall certainty of the result. For glutathione, as compared to the results from older and smaller studies included in the previous Cochrane meta-analysis (80), this analysis pooled results from three recent trials (30, 42, 43) (**Figure 5**), and showed possible benefit with high GRADE certainty. Nonetheless, the selected trials did not fully address existing concerns over glutathione’s effect on oxaliplatin’s clinical efficacy by providing cancer-related outcomes such as overall survival and did not assess the optimal dosage of glutathione. Also, N-3 PUFA was shown by Esfahani et al. (45) to be effective in preventing OIPN when measured in terms of Total Neuropathy Score but further studies are needed to affirm the result given limitations of the study explained in the results section.

In the herbal medicines examined, AC591 and Guilongtongluofang were associated with statistically significant reduction in the risk of OIPN (37, 44). AC591, also known as Huangqi Guizhi Wuwu decoction, is a fixed compound extracted from five Traditional Chinese Medicine (TCM) herbs, while Guilongtongluofang is a concoction extracted from ten TCM herbs. A network meta-analysis on the efficacy of TCM in preventing OIPN also supported the neuroprotective benefit of *Astragalus membranaceus*, the main component in both AC591 and Guilongtongluofang (81). However, there is little evidence on the mechanism, safety profile, and effectiveness of these two compounds when considering the latest oncology guidelines for CIPN (7, 8).

The strengths of this study include the comprehensive coverage of interventions studied between 2005 and 2020, the strict selection criteria for only prospective RCTs where the patients were naïve to neurotoxic chemotherapy, the specificity for chronic OIPN which is distinct from peripheral neuropathy associated with other chemotherapy agents such as cisplatin or paclitaxel, and the holistic rating of interventions using a combination of RR, GRADE score, and risk of bias assessment. A network meta-analysis was also conducted to derive indirect results of the effect of intervention against placebo from studies that compared between two types of interventions.

This study has several limitations common to most meta-analyses. Firstly, it used summary data rather than individual patient data, which limits the ability of the study to control for confounders between trials. Secondly, analysis could only be conducted on trials that were published and searchable *via* English-based databases, which introduces an inherent publication bias. This was mitigated by including conference abstracts in the literature search. Finally, the lack of a standardized assessment method for OIPN limits comparison across trials (82).

### Pre-Clinical Studies and Future Directions

Given the lack of definitive evidence on preventive interventions for OIPN, it is paramount to examine the relevant theoretical background and pre-clinical studies, so as to guide future trials.

Mechanisms for OIPN can be classified into platinum-related or oxaliplatin-specific. Platinum-related mechanisms include direct platinum-induced DNA damage in dorsal root ganglion, mitochondrial dysfunction, oxidative stress, and increased neuronal apoptosis (10, 83). On the other hand, oxaliplatin-specific mechanisms and targeted neuroprotective interventions have been examined in various pre-clinical studies, as summarized in a recent systematic review by Kawashiri et al. (84). Unfortunately, success in animal-based models has not consistently translated into clinical effectiveness. Various agents including anti-oxidants have been shown to reduce oxaliplatin-induced behavior alterations in mice (85), but when assessed in clinical trials, they range from being not recommended [e.g. Vitamin E (α-tocopherol)] to requiring further evidence to validate neuroprotective potential (e.g. glutathione and n-3 PUFA). Nonetheless, there are many emerging therapeutic targets worthy of further clinical assessment. Notable mechanisms in pre-clinical studies include remediation of oxaliplatin-induced peroxisome alterations by activating Peroxisome Proliferator Activated Receptor-γ (PPAR-γ) (86), antagonism of noxious hypersensitivity mediated by interactions between astrocytic vascular endothelial growth factor-A (VEGF-A) and VEGF receptor-1 (87), and alternatively *via* the α9α10 nicotinic acetylcholine receptors (nAChR) (88), and reduction of neuronal inflammation by promoting Sphingosine 1-Phosphate Receptor 2 (S1P_2_) signaling (89).

Lastly, a paucity and need for more studies on non-pharmacological interventions was noted. Only one trial, exploring air wave pressure in preventing OIPN, fulfilled the selection criteria (59), however it was unable to demonstrate any significant benefit. In other CIPN prevention studies, four main non-pharmacological interventions have previously been assessed in RCTs, namely acupuncture (17), exercise (18, 90), compression therapy (91), and cryotherapy (15, 16, 19–21, 92, 93). Of these, the most promising intervention has been cryotherapy (7, 8). In a recent meta-analysis on the effectiveness of cryotherapy by Bailey et al. (94), it was noted that all seven trials assessed taxane-induced peripheral neuropathy, although the trial by Beijers et al. also recruited patients on oxaliplatin (21). Given that cold exposure is generally avoided to prevent acute OIPN, it is interesting that this study explored cryotherapy for CIPN prevention including an oxaliplatin cohort. One-third of the patients in this trial discontinued the intervention due to intolerance, however further details are unavailable. This preliminary evidence suggests future studies can be conducted explore cryotherapy for OIPN prevention.

## Conclusions

In summary, despite the numerous pharmacological interventions reported in trials in recent years, there is insufficient evidence of any clinical success in OIPN prevention. Given the encouraging results seen in pre-clinical studies on OIPN prevention and emergence of non-pharmacological interventions in CIPN prevention, future studies could explore their potential. Moreover, studies with adequate recruitment, randomization and blinding, and standardized reporting of outcomes are necessary.

## Data Availability Statement

The data analyzed in this study is subject to the following licenses/restrictions: Readers may contact the authors for access to our datasets. Requests to access these datasets should be directed to siyu.peng@mohh.com.sg.

## Author Contributions

RS, YS, and AB conceived the research question and supervised the study. SP and AY planned the study methodology, performed data collection, analyzed results, and drafted the manuscript. SP performed statistical analysis with advice from YS. NC drafted and registered the study with the PROSPERO database. All authors provided critical feedback and helped shape the discussion. All authors contributed to the article and approved the submitted version.

## Funding

This work was supported by the National Medical Research Council under its Clinician Scientist – Individual Research Grant (NMRC/CNIG/1167/2017); the National University Health System under its NUHS Summit Research Program - Cancer (NCSP N-171-000-493-001); National University of Singapore under its N.1 Institute for Health’s Translational Core.

## Conflict of Interest

RS is a member on the advisory board of Bristol Myers Squibb, Merck, Eisai, Bayer, Taiho, Novartis, MSD. He has received honoraria for talks from MSD, Eli Lilly, BMS, Roche, Taiho, Astra Zeneca. He has received travel funding from Paxman Coolers Ltd., Roche, Astra Zeneca, Taiho, Eisai. He has received research funding from Paxman Coolers, MSD. AB has received travel funding from Paxman Coolers Ltd.

The remaining authors declare that the research was conducted in the absence of any commercial or financial relationships that could be construed as a potential conflict of interest.

## Publisher’s Note

All claims expressed in this article are solely those of the authors and do not necessarily represent those of their affiliated organizations, or those of the publisher, the editors and the reviewers. Any product that may be evaluated in this article, or claim that may be made by its manufacturer, is not guaranteed or endorsed by the publisher.
