# Supplementary material for: Prevention of Oxaliplatin-Induced Peripheral Neuropathy: A Systematic Review and Meta-Analysis
Source: Front Oncol. 2022 Feb 3;12:731223. doi: 10.3389/fonc.2022.731223 (PMC8853097; doi:10.3389/fonc.2022.731223)
Supplement: Supplementary file 1 [file DataSheet_1.pdf]

Prevention of oxaliplatin-induced peripheral neuropathy: a systematic review and meta-analysis

Siyu Peng (MBBS, MRCP, MMed)[1†], Ariel Fangting Ying (BSc Hons)[2†], Nicholas Jian Hao Chan (MBBS)[1], Raghav Sundar (MBBS, MRCP, MMed, MCI, FAMS)[3,4,5], Yu Yang Soon (MBBS, MSc, FRANZCR)[6*], Aishwarya Bandla (PhD)[3,4*]

Authors' affiliations:

1. Department of Medicine, National University Health System, Singapore.
2. Health Services and System Research, Duke-NUS Medical School, Singapore.
3. Department of Haematology-Oncology, National University Cancer Institute Singapore, National University Health System, Singapore.
4. The N.1 Institute for Health, National University of Singapore, Singapore.
5. Yong Loo Lin School of Medicine, National University of Singapore, Singapore.
6. Department of Radiation Oncology, National University Cancer Institute Singapore, National University Health System, Singapore.

[†]These authors contributed equally to the work.

[*]**Correspondence to:**

Dr. Aishwarya Bandla, The N.1 Institute for Health, National University of Singapore, 28 Medical Drive, Singapore 117456, Singapore.
Email: aishwarya.bandla@nus.edu.sg

Dr. Yu Yang Soon, Department of Radiation Oncology, National University Cancer Institute Singapore, National University Health System, Singapore.
Email: yu_yang_soon@nuhs.edu.sg

Supplementary Methods

Data sources and searches

Two independent researchers (PS and AYF) conducted a comprehensive literature search in three databases: PubMed-MEDLINE, Embase and Scopus without language restrictions. The dates searched were from 1 Jan 2005 to 8 Aug 2020.

Differences in opinion were reconciled through discussion and consultation with an independent third party.

The search terms included

- Neuroprotect* OR chemoprotect* OR prevent* OR protect* OR prophy* OR reduc*
- Oxaliplatin* OR Eloxatin*
- Neuropath* OR neuro* OR nerv*

The following filters were applied to the respective databases

Engine	PubMed (1)	Embase (1)	Scopus (2)
Filter	(randomized controlled trial[pt] OR controlled clinical trial[pt] OR randomized[tiab] OR placebo[tiab] OR drug therapy[sh] OR randomly[tiab] OR trial[tiab] OR groups[tiab] NOT (animals [mh] NOT humans [mh])) OR meta-analysis [pt] OR meta-analys*	'crossover procedure':de OR 'double-blind procedure':de OR 'randomized controlled trial':de OR 'single-blind procedure':de OR (random* OR factorial* OR crossover* OR cross NEXT/1 over* OR placebo* OR doubl* NEAR/1 blind* OR singl* NEAR/1 blind* OR assign* OR allocat* OR volunteer*):de,ab,ti<br><br>NOT ([animals]/lim NOT [humans]/lim)	(INDEXTERMS ("clinical trials" OR "clinical trials as a topic" OR "randomized controlled trial" OR "Randomized Controlled Trials as Topic" OR "controlled clinical trial" OR "Controlled Clinical Trials" OR "random allocation" OR "Double-Blind Method" OR "Single-Blind Method" OR "Cross-Over Studies" OR "Placebos" OR "multicenter study" OR "double blind procedure" OR "single blind procedure" OR "crossover procedure" OR "clinical trial" OR "controlled study" OR "randomization" OR "placebo")) OR (TITLE-ABS-KEY (("clinical trials" OR "clinical trials as a topic" OR "randomized controlled trial" OR "Randomized Controlled Trials as Topic" OR "controlled clinical trial" OR "Controlled Clinical Trials as Topic" OR "random allocation" OR "randomly allocated" OR "allocated randomly" OR "Double-Blind Method" OR "Single-Blind Method" OR "Cross-Over Studies" OR "Placebos" OR "cross-over trial" OR "single blind" OR "double blind" OR "factorial design" OR "factorial trial"))) OR (TITLE-ABS (clinical trial* OR trial* OR rct* OR random* OR blind*))

Quality assessment

We used the approach proposed by the Grading of Recommendations, Assessment, Development and Evaluation (GRADE) Working Group (www.gradeworkinggroup.org) to evaluate the certainty of the result across 4 domains, namely risk of bias, inconsistency, indirectness and imprecision.

The risk of bias was assessed across 6 items in the Risk of Bias (RoB) table in the RevMan 5.3 software, namely "random sequence generation (selection bias)", "allocation concealment (selection bias)", "blinding (performance bias and detection bias)", "incomplete outcome data (attrition bias)" and "selective reporting (reporting bias)". Each bias assessment item was graded as having “High”, “Unclear” or “Low” risk of bias.

The following criteria were applied while translating the risk of bias result from RevMan to GRADE scoring:

- Very serious: more than one “High” risk item from RevMan RoB
- Serious: one “High” risk item or two “Unclear” risk items from RevMan RoB
- Not serious: less than one “High” risk item from RevMan RoB

Data Synthesis and Analysis

Null hypothesis: There is no difference in the risk of oxaliplatin induced peripheral neuropathy (OIPN) between intervention vs placebo.

The primary outcome extracted was the Risk Ratio (RR) of grade 2 and above OIPN assessed using the Common Terminology Criteria for Adverse Events (CTCAE). Duration of assessment follows the longest duration in the trials, except when there is significant drop out rate, in which case an earlier cut off is used to mitigate attrition bias.

If there were two or more studies reporting the risk ratio of OIPN by comparing the same intervention to placebo, we synthesized the overall risk ratio using Forest plots using the random effect model of meta-analysis in RevMan 5.3 Software. We used the Q-test to assess between study heterogeneity, which is presented in terms of I^2 of heterogeneity. If significant methodological or statistical heterogeneity was detected, we performed sensitivity analysis based on factors that could have contributed to heterogeneity.

For continuous outcomes such as pain score, neuropathy score, we calculated the difference in means and expressed the results with 95% confidence intervals (CI).

For outcomes that do not fit the above criteria, we assessed outcomes as they were presented in the original trial, such as various nerve conduction studies results.

Lastly, for studies that compared two types of interventions, we conducted network meta-analysis using R programming to obtain indirect evidence of effect of each type of intervention versus placebo or no additional intervention.

All reported *p*-values are 2-sided. A *p* value of less than 0.05 was considered to indicate statistical significance. All analyses were performed with the RevMan 5.3 software and R software.

A review protocol was created prior to the intervention and registered with PROSPERO (registration number CRD42021225095), with no amendment made since submission.

Supplementary Figures

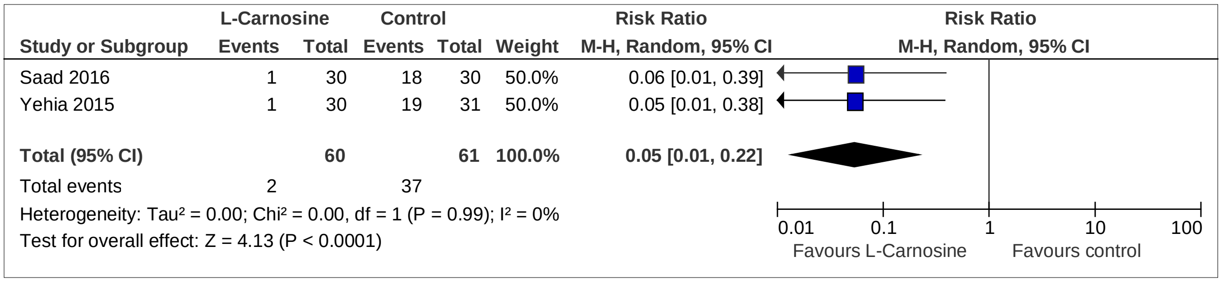

Supplementary Figure 1. Forest plot for comparison of the incidence of OIPN of CTCAE grade ⩾ 2: L-Carnosine versus control (no additional intervention)

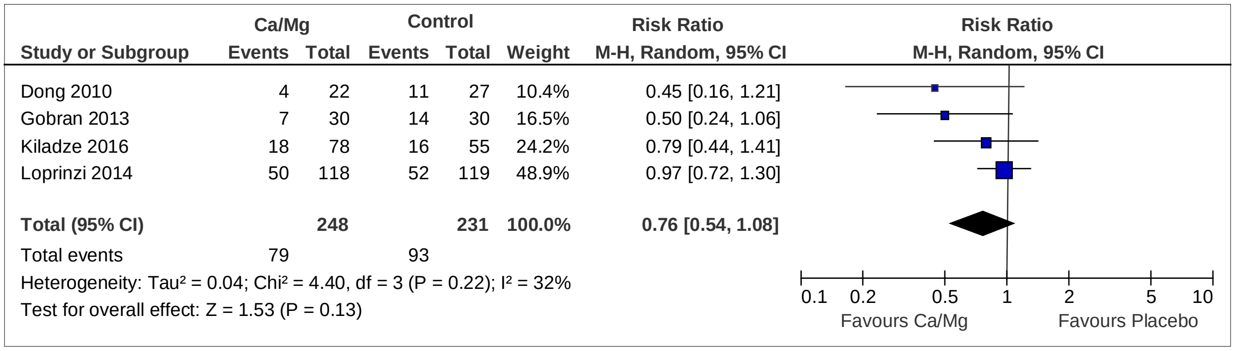

Supplementary Figure 2. Forest plot for comparison of the incidence of OIPN of CTCAE grade ⩾ 2: Ca/Mg versus placebo, sensitivity analysis by excluding trials with early termination

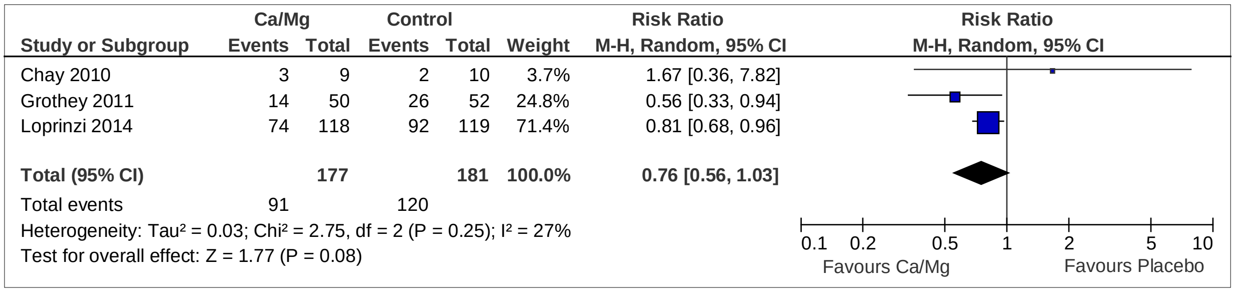

Supplementary Figure 3. Forest plot for comparison of the incidence of OIPN of OSS grade ⩾ 2: Ca/Mg versus placebo

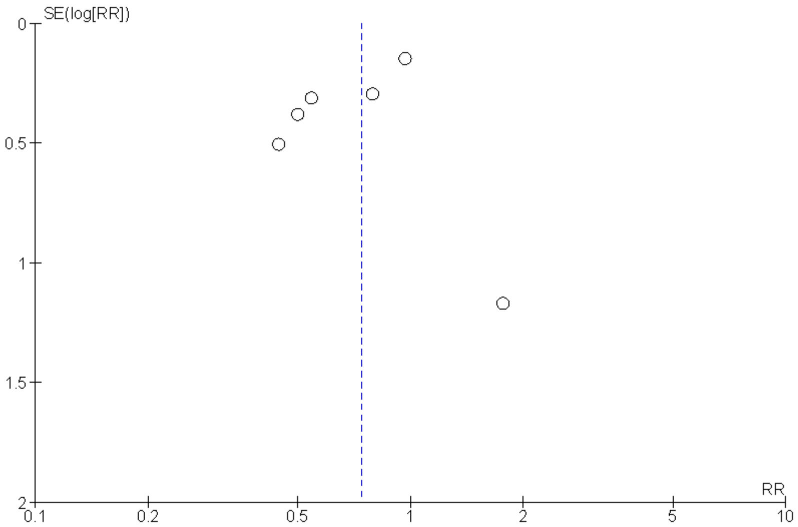

Supplementary Figure 4. Funnel plot of the six trials included in meta-analysis for comparison of the incidence of OIPN of CTCAE grade ⩾ 2 with Ca/Mg versus placebo

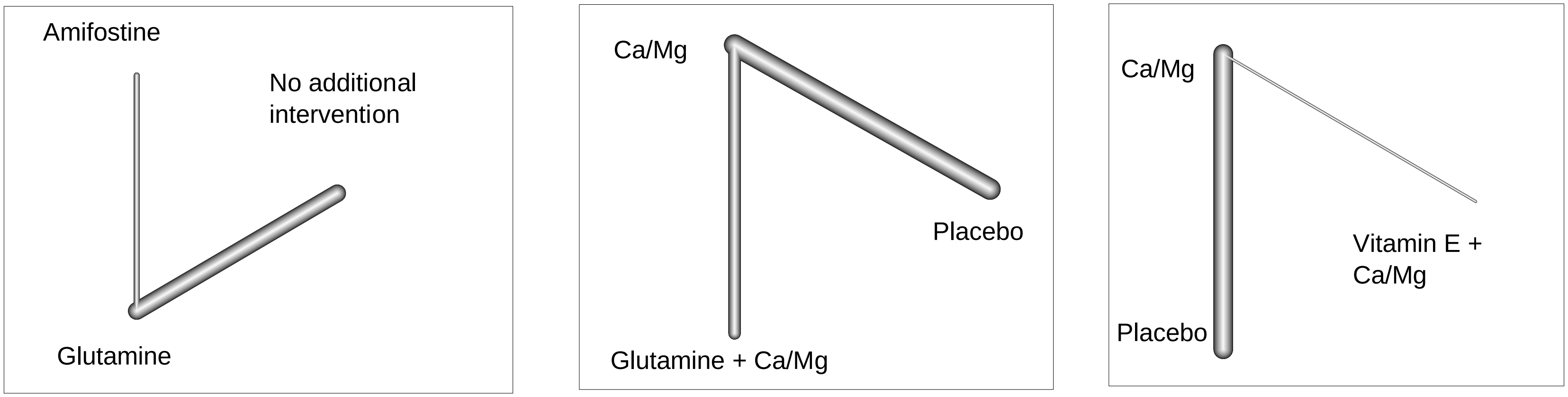

(A) (B) (C)

Supplementary Figure 5. Network diagram comparing (A) Amifostine, Glutamine and no additional intervention, (B) Glutamine + Ca/Mg, Ca/Mg and placebo, and (C) Vitamin E + Ca/Mg, Ca/Mg and placebo.

Network graphs illustrate the type of comparison between different interventions. The presence of lines between a pair of nodes (interventions) indicates direct comparison from original trials, while the absence of line indicates indirect comparison derived from network meta-analysis. The thickness of the lines is proportional to the number of trials that conducted the respective direct comparisons.
